# Triple-Negative Breast Cancer Comparison With Canine Mammary Tumors From Light Microscopy to Molecular Pathology

**DOI:** 10.3389/fonc.2020.563779

**Published:** 2020-11-12

**Authors:** Asadoor Amirkhani Namagerdi, Danila d’Angelo, Francesca Ciani, Carmelina Antonella Iannuzzi, Francesco Napolitano, Luigi Avallone, Michelino De Laurentiis, Antonio Giordano

**Affiliations:** ^1^ Department of Veterinary Medicine and Animal Production, University of Naples Federico II, Naples, Italy; ^2^ Cell Biology and Biotherapy Unit, Istituto Nazionale Tumori- IRCCS- Fondazione G. Pascale, Naples, Italy; ^3^ CCEINGE, Biotecnologie Avanzate, Naples, Italy; ^4^ Breast Oncology Division, Istituto Nazionale Tumori IRCCS Fondazione G. Pascale, Naples, Italy; ^5^ Center for Biotechnology, College of Science and Technology, Sbarro Institute for Cancer Research and Molecular Medicine, Temple University, Philadelphia, PA, United States; ^6^ Department of Medical Biotechnologies, University of Siena, Siena, Italy

**Keywords:** triple-negative breast cancers, canine mammary tumors, tumor biomarkers, hormonal receptors, genetics and epigenetics

## Abstract

Many similar characteristics in human and dog cancers including, spontaneous development, clinical presentation, tumor heterogeneity, disease progression, and response to standard therapies have promoted the approval of this comparative model as an alternative to mice. Breast cancer represents the second most frequent neoplasm in humans after lung cancer. Triple-negative breast cancers (TNBC) constitute around 15% of all cases of breast cancer and do not express estrogen receptor (ER), progesterone receptor (PR), and do not overexpress human epidermal growth factor receptor 2 (HER2). As a result, they do not benefit from hormonal or trastuzumab-based therapy. Patients with TNBC have worse overall survival than patients with non-TNBC. Lehmann and collaborators described six different molecular subtypes of TNBC which further demonstrated its transcriptional heterogeneity. This six TNBC subtype classification has therapeutic implications. Breast cancer is the second most frequent neoplasm in sexually intact female dogs after skin cancer. Canine mammary tumors are a naturally occurring heterogeneous group of cancers that have several features in common with human breast cancer (HBC). These similarities include etiology, signaling pathway activation, and histological classification. Molecularly CMTs are more like TNBCs, and therefore dogs are powerful spontaneous models of cancer to test new therapeutic approaches, particularly for human TNBCs. More malignant tumors of the breast are more often ER and PR negative in both humans and dogs. Promising breast cancer biomarkers in both humans and canines are cancer-associated stroma (CAS), circulating tumor cells and tumor DNA (ctDNA), exosomes and miRNAs, and metabolites.

## Introduction 

Breast cancer comprises the second most common neoplasm in humans after lung cancer (1). The human breast consists of a branching ductal network consisting of an inner layer of polarized luminal epithelial cells, and an outer layer of myoepithelial cells. The ductal network terminates in lobular units commonly called the terminal duct lobular units (TDLUs) (2). TDLUs produce milk and are the primary source of most breast cancer precursors and cancers (3). Normal breast development and mammary stem cells are regulated by some signaling pathways, such as estrogen receptors (ERs), HER2, and Wnt/b-catenin signaling pathways, that influence stem cell proliferation, cell death, cell differentiation, and cell motility (4). Crosstalk between epithelial and stromal cells is important for the normal development and differentiation of the mammary gland. Tumors consist not only of neoplastic cells but also present a substantially changed surrounding stroma. The tumor microenvironment (TME) or cancer-associated stroma (CAS) is identified as a crucial element for tumor development and progression, as well as a measurable parameter of response to treatment (5). In breast carcinoma if the tumor is confined to the epithelial component it is called “in-situ carcinoma” and if it invades the stroma it is called invasive carcinoma. It is also important to know if the tumor has arisen from the duct (ductal carcinoma) or the lobule (lobular carcinoma) (6). In 2012 the name for the most common type of breast cancer was changed from invasive ductal carcinoma, noswise specified (NOS) (2003) to invasive carcinoma of no special type (NST) (7). Special types of breast cancer account for up to 25% of all breast cancers. The latest edition of the World Health Organization recognizes at least 17 different histological special types (8). Tumor size, lymph node involvement, histologic type, histologic grade, and a receptor (estrogen receptor (ER), progesterone receptor (PR), human epidermal growth factor receptor 2 (HER2)) expression status by immunohistochemistry (IHC) have been well established as prognostic and predictive factors for breast cancers (9).

Triple-negative breast cancer (TNBC) constitutes around 15% of all breast cancer cases and is characterized by tumors that do not express estrogen receptor (ER), progesterone receptor (PR), and do not overexpress human epidermal growth factor receptor 2 (HER2) (10). As a result they do not benefit from hormonal or trastuzumab-based therapy (11). Patients with TNBC have worse overall survival than patients with non-TNBC (12). They typically occur at a younger age, with higher histologic grade, larger size, high rate of p53 mutations, and Ki-67 staining and generate local and visceral metastases rather than bone metastases (13). Treatment options are limited to surgery, radiotherapy chemotherapy (14). Most patients receive adjuvant anthracyclines (doxorubicin or epirubicin), taxanes (docetaxel), and an alkylating agent (cyclophosphamide) (15). Despite receiving standard anthracycline taxane-based chemotherapy 30%–40% of patients with early-stage TNBC develop metastatic disease and die of cancer. Therefore, TNBC patients strongly require new therapies (16). Some of the novel therapies for TNBC include poly-ADP ribose polymerase (PARP) inhibitors, platinum salts, non-taxane microtubule-stabilizing agents, anti-VEGF monoclonal antibody, inhibitors of EGFR/P13K/AKT/mTOR signaling pathways, androgen receptor inhibitor, histone deacetylase inhibitor, immunotherapies, vaccines, and inhibitors of Hedgehog, NOTCH and WNT/β-catenin signaling pathways (17).

Domestic dogs (Canis lupus familiaris) are excellent models of human complicated diseases for many reasons, including their approachability and popular role in different cultures (18). Many similar characteristics in human and dog cancers including, spontaneous development, clinical presentation, tumor heterogeneity, disease progression, and response to standard therapies have promoted the approval of this comparative model as an alternative to mice. Breast cancer represents the second most frequent neoplasm in sexually intact female dogs after skin cancer (1). Canine mammary tumors are a naturally occurring heterogenous group of cancers that have several features in common with human breast cancer. These similarities include etiology, signaling pathway activation and histological classification (19). Bitches typically have five pairs of mammary glands, which are called thoracic (2 pairs), abdominal (2 pairs), and inguinal glands (1 pair) (20). The appearance of canine mammary tumors (CMT) in bitches under the age of two is rare but increases remarkably for bitches over six years old (21). Old age, mixed breed, and large size lead to its development and reflect malignancy risk factors (22). Maiti et al. (23) studied 70 cases of CMTs. Forty-eight cases had solitary growth and 36 tumors were pedunculated and 34 were sessile. Thirty-eight mammary growths were ulcerated and inflamed and the remaining 32 were intact and subcutaneous. The most affected mammary glands were caudal abdominal and inguinal (4th and 5th). The majority of mammary gland tumors in female dogs are of epithelial origin, and approximately 50% are malignant (24). The incidence of CMT in female dogs spayed before their first heat is 0.05% but increases to 8% or 26% if spayed after the first or second heat, respectively (25). Several studies have shown that some breeds have a genetic tendency to suffer from CMT including Miniature Poodles, Dachshunds, Maltese, Yorkshire Terriers, Cocker Spaniels, and German Shepherds (26). Dogs as models for human cancer present the potential of overcoming limitations of xenograft and genetically engineered rodent models resulting in a greater understanding of tumor biology and the discovery of biomarkers. It is notable that larger tumor size, the presence of lymph node metastases, and advanced clinical stage are associated with worse prognosis in both species (27). Mastectomy is the treatment of choice for the mammary tumors in dogs (28), and adjuvant chemotherapy is recommended for CMTs with regional or distant metastases and poor-prognosis mammary tumors (29). More malignant tumors of the breast are more often ER and PR negative in both humans and dogs (30). Histological types, cell lines, molecular classification, genetic and epigenetic heterogeneities, and tumor biomarkers will be discussed in TNBCs and CMTs in the following paragraphs.

## TNBC and CMT Histologic Heterogeneity

In humans all studies reported invasive ductal carcinomas of not otherwise specified (NOS) to be the predominant histologic subtype seen within all the patient populations, with invasive lobular carcinoma being the subsequent predominant histologic subtype (31). The special histologic types that are commonly triple-negative (TN) include, Carcinoma with medullary features, Carcinoma with apocrine differentiation, Metaplastic breast carcinoma, Acinic cell carcinoma, Adenoid cystic carcinoma, and Secretory carcinoma. **Table 1** shows the percentage of the special histologic types of breast cancer that are triple-negative (32). Invasive ductal carcinomas of no special type display pushing invasive borders, marked degrees of nuclear pleomorphism, lack of tubule formation, and high mitotic rates (33). Invasive lobular carcinoma shows the proliferation of scattered discohesive small cells or tumor cells arranged in a single-file pattern and the round nuclei with scant mitotic figures (34). The histopathologic characteristics of medullary carcinoma include lymphoplasmacytic infiltration, noninvasive microscopic circumscription, syncytial growth pattern >75%, and grade 2 or 3 nuclei (35). Adenoid cystic carcinoma shows myoepithelial differentiation and is characterized by the presence of a dual population of basaloid and luminal cells arranged in various growth patterns like cribriform, glandular, trabecular, or solid (36). Morphologically secretory carcinoma displays tubular, solid, and/or microcystic growth patterns with intra- and extra-cellular dense eosinophilic secretions (37). Acinic cell carcinomas of the breast are solid and can be poorly circumscribed and infiltrating composed of cells, characterized by central round nuclei with prominent nucleoli, and abundant granular, eosinophilic to amphophilic cytoplasm (38). In carcinoma with apocrine differentiation microscopically, the cells are characterized by the typical apocrine features of abundant eosinophilic granular cytoplasm and prominent, and often multiple, nucleoli (39). Metaplastic breast carcinoma is a poorly differentiated heterogeneous tumor that is comprised of a mixture of ductal carcinoma cells with spindle, squamous, chondroid, or osseous elements (40).

**Table 1:** Special histologic types of breast cancer that are commonly triple negative.

Histologic type	Percentage of triple negative tumors
Carcinoma with medullary features	64%–100%
Carcinoma with apocrine differentiation	38%–90%
Metaplastic breast carcinoma	85%–94%
Acinic cell carcinoma	80%–100%
Adenoid cystic carcinoma	85%–100%
Secretory carcinoma	65%–100%

**Figure 1:**
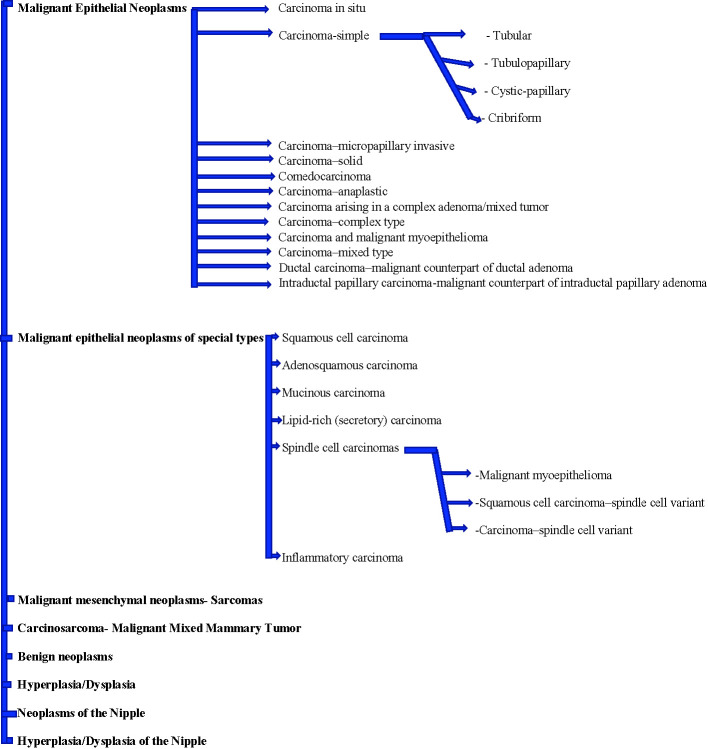
Goldschmidt et al. (41) proposed histological classification of canine mammary tumors (CMT) subtypes.

In 2011, Goldschmidt et al. (41) proposed a new comprehensive histological classification of CMT subtypes based on the classifications previously published by the World Health Organization in 1974 and 1999. Histologically, CMTs are classified as malignant epithelial neoplasms, malignant epithelial neoplasms of special types (Squamous cell carcinoma, Adenosquamous carcinoma, Mucinous carcinoma, Lipid-rich (secretory) carcinoma, Spindle cell carcinoma, and inflammatory carcinoma), malignant mesenchymal neoplasms, carcinosarcoma, benign neoplasms, Hyperplasia/Dysplasia, Neoplasms of the Nipple, and Hyperplasia/Dysplasia of the Nipple (42). Canine mammary malignant epithelial neoplasms by themselves include Carcinoma–in situ, Carcinoma–simple (Tubular, Tubulo-papillary, Cystic-papillary, Cribriform), Carcinoma–micropapillary invasive, Carcinoma–solid, Comedocarcinoma, Carcinoma–anaplastic, Carcinoma arising in a complex adenoma/mixed tumor, Carcinoma–complex type, Carcinoma and malignant myoepithelioma, Carcinoma–mixed type, Ductal carcinoma–malignant counterpart of ductal adenoma, Intraductal papillary carcinoma–malignant counterpart of intraductal papillary adenoma (42) (**Figure 1**). The gross morphology of 26 cases of CMTs was studied by Patel et al. (43). The weight of the tumors varied from 30 to 2000 grams and most of them were round to oval in shape with a soft to hard consistency and a grayish-white cut surface. Of the 229 CMT tumors studied by Goldschmidt et al. (41), 169 (74%) were malignant and 60 (26%) were benign. Among the malignant tumors, complex carcinoma was the most common (13.6%), followed by Carcinoma and malignant myoepithelioma (11.8%), Solid carcinoma (11.8%), Anaplastic carcinoma (10.6%), Comedocarcinoma (10%), Simple tubular carcinoma (8.9%), Carcinoma arising in benign mixed tumor (8.3%), Simple tubulopapillary carcinoma (7.1%), Intraductal papillary carcinoma (7.1%), Adenosquamous carcinoma (5.9%), and Carcinosarcoma (4.7%). Myoepithelial cell proliferation is much more common, occurring in more than 20% of CMTs compared to less than 0.1% in HBCs. Canine simple carcinomas have no myoepithelial cell proliferation, while canine complex carcinomas have both proliferating luminal and myoepithelial cells. Histologically, canine simple carcinomas mirror human breast carcinomas (44).

## Molecular Classification of TNBC, CMT, and Cell Lines

Sørlie et al. (45) by using gene expression profiling (GEP) on 456 cDNA clones of the breast, classified human breast cancers into five intrinsic subtypes, i.e., luminal A (ER+, PR+, HER2-, KI67-), luminal B (ER+, PR+, HER2+/-, KI67+), HER2 over-expression (ER-, PR-, HER2+), basal-like (ER-, PR-, HER2-, CK5/6+) and normal-like tumors (ER+, PR+, HER2-, KI67-). Normal-like and luminal A tumors have the same immunohistochemistry features but differ on expression pattern (46). Some studies show that normal-like may be an artifact of contamination of tumor RNA with RNA from normal breast cells (47) and normal-like breast cancer subtype is now less frequently used (48) (**Table 2**). In 2007, Herschkowitz et al. (49) identified a new molecular subtype, referred to as claudin-low. Despite the apparent similarity to basal-like tumors, Prat et al. (50) showed that claudin-low tumors as a group did not show high expression of proliferation genes and thus are likely slower-cycling tumors. Clinically, most claudin-low tumors are estrogen receptor (ER)-negative, progesterone receptor (PR)-negative, and epidermal growth factor receptor 2 (HER2)-negative (triple-negative) with poor prognosis. TNBCs and basal-like breast cancers were previously thought to be the same but transcriptomic analyses have shown that TNBCs are highly heterogenous (37). Not all triple negative (TN) tumors are identified as basal-like by gene expression, and not all basal-like tumors are TN (51). In 2011 by using gene expression analyses from 386 tumors, Lehmann et al. (52) described six different TNBC subtypes, including two basal-like (BL1 and BL2), an immunomodulatory (IM), a mesenchymal (M), a mesenchymal stem-like (MSL), and a luminal AR (LAR) subtype. It was shown later that such TNBC subtypes were closely linked to histological types; IM tumors overlapped with medullary breast cancer, M and MSL tumors with metaplastic breast cancer, and LAR tumors with apocrine tumors (4). The BL1 subtype is characterized by an abundance of the components and pathways of cell cycle and cell division. The BL2 subtype involves growth factor signaling pathways (EGF, NGF, MET, Wnt/β-catenin, and IGF1R) as well as glycolysis and gluconeogenesis (53). The IM subtype is identified by expression of genes encoding immune antigens and cytokines, and LAR subtype by androgen receptor signaling (54). The genes included in motility, extracellular matrix, cell differentiation pathways, and epithelial-to-mesenchymal transition (EMT) genes characterize both the M and MSL subtypes, however, the MSL subtype differs in that it expresses low levels of proliferation genes and is enriched for genes associated with mesenchymal stem cells (55). Lehmann et al. (55) demonstrated that the overwhelming majority of tumors classified as claudin-low are composed of M and MSL TNBC subtypes, according to the high levels of EMT-associated genes. Several molecular assays are currently used in the clinical assessment of breast cancer, including MammaPrint, Oncotype DX, PAM50, and Breast Cancer Index (56). Lehmann et al. (52) showed that the six TNBC subtypes displayed various sensitivities to different agents. Their data suggest that patients with basal-like TNBC should receive PARP inhibitors and cisplatin, and patients with the LAR subtype should acquire bicalutamide alone or in combination with PI3K inhibitors, and those with MSL subtype should receive an Src antagonist and a PI3K inhibitor. Masuda et al. (57) showed that after neoadjuvant chemotherapy of 130 TNBC patients with different subtypes, the BL1 subtype had the highest pathological complete response (pCR) rate (52%), and BL2 and LAR had the lowest. Their findings suggest that we especially need to characterize the BL2 and LAR subtypes to implement specific treatment strategies for them.

**Table 2:** Intrinsic subtypes of breast cancer by Sørlie et al. (45).

Intrinsic subtype	IHC status
Luminal A	ER+, PR+, HER2-, KI67-
Luminal B	ER+, PR+, HER2+/-, KI67+
HER2 over-expression	ER-, PR-, HER2+
Basal-like	ER-, PR-, HER2-, CK5/6+

In 2018 He et al. (58) identified three distinct subtypes of TNBC based on immune signatures and named them Immunity High (Immunity-H), Immunity Medium (Immunity-M), and Immunity Low (Immunity-L). They identified high rates of infiltration of cytotoxic T cells and B cells in Immunity-H as compared to the Immunity-L subtype (**Table 3**).

**Table 3:** Molecular subtypes of triple-negative breast cancers (TNBC) by Lehman et al. (42) and He et al. (47).

TNBC subtypes and their genetic abnormalities
**Lehmann et al. classification**
1- Basal-like 1 (BL1) (enrichment of cell cycle and cell division components and pathways)
2- Basal-like 2 (BL2) (growth factor signaling pathways, glycolysis, and gluconeogenesis)
3- Immunomodulatory (IM) (expression of genes encoding immune antigens and cytokines)
4- Mesenchymal (M) (genes included in motility, extracellular matrix, cell differentiation pathways, and epithelial-to-mesenchymal transition (EMT) genes)
5- Mesenchymal stem-like (MSL) (like M subtype and expression of low levels of proliferation genes and high levels of mesenchymal stem cell genes only in MSL subtype)
6- Luminal Androgen Receptor (LAR) (androgen receptor signaling)
**He et al. classification** Immunity high Immunity medium Immunity low (high rates of infiltration of cytotoxic T cells and B cells in Immunity-H compared with Immunity-L)

In 2019, Gruosso et al. (59) identified distinct tumor immune microenvironment (TIME) subtypes characterized by spatial patterns of CD8+ T cell localization and gene expression signatures in therapy-naive TNBC tumors. They identified core CD8 low (corCD8^lo^) and core CD8 high (corCD8^hi^) groups and then CorCD8^lo^ (low infiltration into tumor core) group was classified into immune desert (ID) and margin restricted (MR) and corCD8^hi^ (high infiltration into tumor core) into stroma restricted (SR) and fully inflamed (FI) subgroups. They established TIME classification including ID, MR, SR, and FI subtypes. In line with the TNBC subtypes described by Lehmann et al., they found that corCD8hi tumors were markedly enriched in the immunomodulatory subtype of TNBC. In contrast, corCD8lo tumors were greatly enriched in the mesenchymal subtype.

In the largest canine mammary cancer cohort reported by Abadie et al. (60) 350 female canine mammary cancers were classified as luminal A (14.3%), luminal B (9.4%), no HER2-overexpressing and triple-negative (76.3%) either of the basal-like type (ER- and PR-, EGFR and/or CK5/6+) (58.6%) or of the non-basal-like type (ER- and PR-, EGFR, and CK5/6-) (17.7%) (60). Dogs are therefore powerful spontaneous models of cancer to test new therapeutic approaches, particularly for human triple-negative breast cancers. Im et al. (61) showed that Carcinoma-tubular and carcinoma arising in a complex adenoma/mixed tumor were frequently categorized as luminal A, whereas carcinoma-solid was frequently categorized as basal-like.

Human cancer cell (HCC) lines are a useful tool for researching genetics, molecular biology, biology, and cancer therapy in many types of tumors, including breast cancer (62). HCC 1937 and MDA-MB-468 cell lines are BL1; HCC 1806 and HDQ-P1 are BL2; DU-4475 is IM; BT-549 is M; HS578T, MDA-MB-436, and MDA-MB-231 are MSL; and MDA-MB-453 and CAL-148 are LAR subtypes of TNBC cell lines. AU-565, T-47D, SKBR-3, MDA-MB-361 and MCF-7 are non-TNBC cell lines (63). Zhang et al. (64) developed CMT-7364 a novel triple negative canine mammary cancer cell line that can be used as a promising model for the immunotherapy research and epithelial-mesenchymal transition (EMT) mechanism of TNBC in both canine and humans. Other canine mammary cancer cell lines that have been developed by now include REM 134 mammary carcinoma cell line (65), CMT-1, CMT-2, CMT-3, CMT-4, CMT-5, CMT-6 (66), CMT12, CMT27 (67) and CMT-U27 (ductal invasive carcinoma), CMT-U111 (lobular invasive carcinoma), CMT-U155 (noninvasive ductal carcinoma), CMT-U131 (infiltrating ductal carcinoma of scirrhous type) and CMT-U229 (atypical benign mixed tumor) (68). Breast cancer cell lines can be studied for the expression of different genes and proteins for revealing mutations and investigated for molecular characterization of receptors and cellular pathways by omics methods (69) (**Table 4**).

**Table 4:** Triple-negative breast cancers (TNBC) and canine mammary tumors (CMT) cell lines.

TNBC cell lines	CMT Cell lines
TNBC (BL 1): HCC 1937, MDA-MB-468	Triple negative: CMT-7364
TNBC (BL 2): HCC 1806, HDQ-P1	Ductal invasive carcinoma: CMT-U27
TNBC (IM): HCC 1806, HDQ-P1	Lobular invasive carcinoma: CMT-U111
TNBC (M): BT-549	Noninvasive ductal carcinoma: CMT-U155
TNBC (MSL): HS578T, MDA-MB-436, MDA-MB-231	Infiltrating ductal carcinoma of scirrhous type: CMT-U131
TNBC (LAR): MDA-MB-453, CAL-148	Atypical benign mixed tumor: CMT-U229
Non-TNBC: AU-565, T-47D, SKBR-3, MDA-MB-361, MCF-7	

## Genetic Heterogeneity and Epigenetics of TNBC and CMTs

Genes with at least a fourfold rise in pathogenic mutations in BC cases compared with unaffected controls are usually categorized as high-risk BC genes. These include BRCA1/2, CDH1, PALB2, PTEN, STK11, and TP53 (70). While germline BRCA1/2 mutations occur in 5.3% of unselected breast cancers according to The Cancer Genome Atlas (TCGA), a recent study showed that 11.2% of unselected TNBC cases had deletions in the BRCA1 (8.5%) and BRCA2 (2.7%) respectively (71). About 70% of breast cancers in BRCA1 mutation carriers and up to 23% of BRCA2 carriers are triple-negative (72). The somatic mutation landscape of TNBC shows the highest frequency of TP53 mutations, up to 80% (73). Shah et al. (74) sequenced 104 TNBC tumors and found that the most common mutation was TP53 mutation (53.8%), followed by PIK3CA mutations (10.7%). Genomic changes in the RB1 gene are relatively common in TNBCs and loss of Rb protein expression is seen in more than 40% of cases (75). One of the most commonly found genetic modifications in TNBC is the amplification of the MYC gene which is identified in more than 60% of samples (76). Gains in chromosomes 1q, 8q, 17q, 20q, and losses in 5q, 6q, 8p are common in breast cancer. Estrogen receptor (ER)-negative cancers frequently harbor losses in 5q and gains in 6p compared with hormone receptor-positive cancers (77). Secretory breast carcinoma is the only epithelial tumor of the breast with a t (12;15) balanced translocation that makes an ETV6-NTRK3 gene fusion and encodes a chimeric tyrosine kinase (78). Like salivary gland ACC, the breast adenoid cystic carcinoma shows the t (6;9) translocation leading to the development of MYB-NFIB gene fusion and immunopositivity for MYB by immunohistochemistry (IHC) (79). High-resolution copy number alteration (CNA) profile in TNBC showed that the most common gains of the entire chromosome arms included 1q, 8q, 10p and 12p, and losses of 5q, 8p, and 17p, and the most frequent focal gains were narrowed down to 3q and 19q and focal losses were identified most often in 3q and 12q (80). In TNBC, from the genomic loss-of-heterozygosity (LOH) landscape, the most frequent genes exhibiting LOH associated monoallelic expression (MAE) were found within chromosomes 3p, 5q, 8p, 10p, 14, and 17 (81). The heritable and reversible epigenetic mechanisms include changes in DNA methylation, histone modifications, and small noncoding microRNAs (miRNA) (82). Histones can be changed to influence gene expression in many ways, including acetylation, phosphorylation, methylation, ubiquitylation, and sumoylation (83). Some genes have been commonly reported to be methylated in breast cancer including RASSF1A, ERα, PR, RARβ, CCND2, and PITX2. A study showed that the methylation profile of TN tumors is distinguished by the methylation of 5 genes (CDKN2B, CD44, MGMT, RB and p73) and the non-methylation of 11 genes (GSTP1, PMS2, MSH2, MLH1, MSH3, MSH6, DLC1, CACNA1A, CACNA1G, TWIST1, and ID4) (84). Methylation of the BRCA1 promoter is frequent in triple-negative breast cancers (TNBC) and leads to a tumor phenotype similar to BRCA1-mutated tumors (85). The phenotype that some sporadic tumors share traits with familial-BRCA cancer is called BRCAness (86). This particular status may be due to the hypermethylation of the promoter region of the BRCA1 gene (87). There are three loci in human chromosome 9p21 as tumor suppressor genes including, CDKN2A (p16INK4a), CDKN2A (p14ARF), and CDKN2B (p15INK4b) (88). The CDKN2A locus controls the p16INK4a/CDK4/pRb pathway and p14ARF/p53 pathway (89). The region of the human chromosome, 9p21 encompassing the CDKN2B/CDKN2A or INK4A/ARF/INK4B gene locus, corresponds to regions of dog chromosome 11, mouse chromosome 4, and rat chromosome 5. These regions have been demonstrated to be frequently mutated in various types of cancer (90). Oncogenic pathways and accompanying genes, such as PI3K/AKT, KRAS, MAPK, Wnt, β-catenin, BRCA2, ESR1, and P-cadherin, are generally up-regulated while tumor-suppressive pathways, such as p53, p16/INK4A, PTEN, and E-cadherin, are down-regulated in human and canine breast cancer (1). Loss of the E-cadherin expression is a characteristic of epithelial-mesenchymal transition (EMT). A worse prognosis is associated with low expression of E-cadherin (evaluated by IHC in tumor tissues) in both human and animal patients but it should also be analyzed with other biomarkers, such as Ki67 (26). Several of the human cancer predisposition genes are present in the constitutional (germline) DNA of dogs with cancer; for example, BRCA1/BRCA2 and TP53 germline mutations. In humans, germline mutations of these genes cause hereditary breast and ovarian cancer syndrome and Li–Fraumeni syndrome, respectively (91). Liu et al. (44) performed whole-genome sequencing, whole-exome sequencing (WES), RNA-seq and/or high-density arrays on 12 CMTs, including seven simple carcinomas and four complex carcinomas and demonstrated that the possibility that canine simple carcinomas emerge from genomic aberrations while complex carcinomas emerge from epigenomic alterations. Many of the genomic aberrations in canine simple carcinomas accurately reiterate main features of human breast cancer.

CAS comprises various cell types such as fibroblasts, leukocytes, adipocytes, and myoepithelial and endothelial cells and includes extracellular matrix (ECM), soluble factors such as cytokines, hormones, growth factors and enzymes, and physical properties as pH and oxygen content (5). Amini et al. (92) developed a protocol for laser-capture microdissection (LCM) on formalin-fixed paraffin-embedded (FFPE) tissue sections of clinical mammary carcinomas in order to precisely isolate RNA from CAS and normal stroma from FFPE tissue sections of 13 canine simple mammary carcinomas. After RNA extraction, quality control, quantitation, and preamplification, the relative mRNA levels of selected genes were measured by RT-qPCR. Amini et al. (93) confirmed differential expression in CAS compared to the normal stroma of three genes including upregulation of α-smooth muscle actin (α-SMA, encoded by ACTA2), upregulation of collagen 4α1 (COL4A1) and downregulation of vimentin in CAS on protein level utilizing immunofluorescence.

Markkanen et al. (94) analyzed CAS and normal stroma from 15 clinical cases using their laser capture microdissection (LCM) coupled with RNA-seq (LCMRNAseq) pipeline to identify stromal reprogramming in canine simple mammary carcinoma on a transcriptome-wide scale. They revealed strong increases in mesenchymal stem cells, gamma delta T-cells, macrophages, plasmoid dendritic cells, and natural killer T-cells in CAS and demonstrated that commonly deranged pathways between canine and human CAS included angiogenesis, epithelial-mesenchymal transition, glycolysis, and immune response pathways. In order to analyze transcriptional reprogramming of adenoma associated stroma (AAS) of 13 canine mammary adenomas compared to previous data from 15 canine mammary carcinomas, Amini et al. (95) applied weighted gene co-expression network analysis (WGCNA) and identified six clusters of highly positively correlated genes and subsequently identified four potentially interesting modules including blue, brown, turquoise and yellow modules. They showed that TGFbeta signaling, glycolysis, mitotic spindle, epithelial to mesenchymal transition, mTORC1 signaling, unfolded protein response, apical surface, interferon-gamma response and G2M checkpoint demonstrated greatly increased enrichment only in CAS and pathways involving pancreas beta cells, fatty acid metabolism, spermatogenesis, heme metabolism and IL2-STAT5 signaling showing dramatically reduced enrichment only in CAS. Markkanen et al. (94) ranked the samples in The Cancer Genome Atlas (TCGA) breast cancer subset (that contains >1000 human tumor samples) analogous to the stromal enrichment scores to compare their canine-derived stromal signature and found that the canine-derived stromal signature was strongly positively associated with the enrichment of human-derived stromal signature of the TCGA breast cancer subset.

## Biomarkers, TNBC and CMTs (Tumor-Associated Biomarkers, Cancer-Associated Stroma, Circulating Tumor Cells and Tumor DNA, Exosomes, miRNAs, Proteomics and Metabolomics)

Biomarkers include genes and genetic variations, differences in messenger RNA (mRNA) and/or protein expression, posttranslational modifications of proteins, and metabolite levels (96). The principal biomarkers, which are usually immunohistochemically tested on breast surgical specimens, include ER and PR, Mib1/Ki-67, and HER2/neu expression (97). The estrogen receptor (ER) is a transcription factor that regulates events of gene expression resulting in cell division (98). Estrogen receptors (ER) include ER-alpha, ER-beta, and a new membrane receptor G protein-coupled receptor 30 (GPR30) (99). About 75% of breast cancer cases are ERα positive at diagnosis (100). A member of the nuclear receptor family, the progesterone receptor is a well-known, estrogen receptor (ER)-regulated gene that is expressed in more than two-thirds of ER-positive breast cancers (101). The human epidermal receptor protein-2 (c-erbB-2; HER2) oncogene protein is a transmembrane glycoprotein in the epidermal growth factor receptor family (102). IHC is the most commonly used method of assessing these factors, although fluorescent *in situ* hybridization (FISH) also has a prominent role in HER2 testing (103). The American Society of Clinical Oncology (ASCO), and the College of American Pathologists (CAP) recommend that ER and PR assays be considered positive if the sample contains at least 1% positive tumor nuclei (fixed in 10% neutral buffered formalin) using the IHC method (104). ASCO/CAP guidelines, recognize tumors as amplified if the HER2/CEP17 ratio (R) is more than 2.2 or, the absolute HER2 copy number (CN) exceeds 6 in the absence of CEP17 assessments (105). The TNBC is a subtype of breast cancer that lacks steroid receptors, i.e., estrogen and progesterone receptors, and does not overexpress the HER2 gene (106). Several pathological biomarkers are used to identify subgroups of TNBC, including P53, cytokeratin (CK) 5/6, CK14, epidermal growth factor receptor (EGFR), and Ki-67 (107). Ki-67 is a nuclear DNA-binding protein expressed in proliferating mammalian cells (108). It is expressed in cell cycle phase S, G1, G2, and M in the cell nucleus reaching a peak during mitosis. The Ki-67 index is relatively higher in TNBC than in non-TNBC (107). The human epidermal growth factor receptor (HER) family includes the epidermal growth factor receptor (EGFR), HER2 (erbB2/neu), HER3 (erbB3), and HER4 (erbB4) (109). It has been reported that at least 50% of TNBC cases have gene amplification or high expression levels of EGFR (110). Keratins (Cytokeratins) are the intermediate filament (IF)-forming proteins of epithelial cells (111). 2-D isoelectric focusing and SDS-PAGE were used by Mole et al. (1982) to map the keratin profiles of normal human epithelia, tumors, and cultured cells. They grouped keratins into two types, basic to neutral type II keratins as K1-K8 and acidic type I keratins as K9-K19 (112). Genome analyses have recently identified that humans have 54 functional keratin genes, i.e., 28 types I and 26 types II keratins, and form two clusters of 27 genes each on chromosomes 17q21.2 and 12q13.13 (111). Breast ducts are composed of two types of epithelial cells, the inner luminal cells, and the outer basal/myoepithelial cells. Cytokeratins (CKs) 8 and 18 are expressed in the luminal layer, whereas the basal epithelial layer is characterized by CK5/14 and the transcription factor p63 (113). Basal-like breast cancers typically express basal cytokeratins such as CK5/6, CK14, and CK17. CK5/6 is the most important and relevant marker for defining the basal subgroup of TNBC (114). c-Kit, a type III receptor tyrosine kinase (RTK), plays a pivotal role in cancer occurrence (115). Loss of c-KIT expression in breast cancer is related to malignant transformation of breast epithelium and performed by KIT gene promoter DNA hypermethylation (116). Poly (ADP-ribose) polymerases (PARPs) are a family of related enzymes that share the ability to catalyze the transfer of ADP-ribose to target proteins (117). PARPs constitute a large family of 18 proteins encoded by different genes (118). PARP1 is the most abundant of different PARP isoforms and represents more than 90% of PARP’s catalytic activity in the cell nucleus (119). As homologous recombination repair (HRR) pathway is impaired in BRCA1-mutated tumor cells, PARP inhibition in these cells can lead to the accumulation of DNA damage and ultimately induce cell death because of impaired DNA damage repair (DDR) from both base-excision repair (BER) and HRR dysfunctions (120). The androgen receptor (AR) is a nuclear receptor belonging to the steroid hormone group including also the estrogen receptor (ER), glucocorticoid receptor (GR), progesterone receptor (PR), and mineralocorticoid receptor (MR) (121). AR seems to play a major role in TNBC carcinogenesis (122). AR has appeared as a possible therapeutic target for AR-positive triple-negative breast cancer (TNBC) (123). Programmed cell death protein-1 (PD-1) and programmed death-ligand 1 (PD-L1) are considered as immune checkpoint factors that inhibit the immune reaction to cancer cells (124). It is assumed that TNBC has a relatively high expression of PD-L1, mainly in inflammatory (immune) cells and sometimes in cancer cells (125). The signaling pathway of vascular Endothelial Growth Factor (VEGF) is considered important in the pathophysiology of TNBC. Intratumor and serum levels of VEGF are significantly higher in TNBC compared to non-TNBC (126). The E-cadherin protein (encoded by the CDH1 gene) is normally expressed in breast epithelial tissue and acts as a crucial component of epithelial cell adhesion and epithelial-to-mesenchymal transition (EMT) (127). Loss of membranous expression of E-cadherin is the defining immunohistochemical feature of lobular differentiation in breast carcinoma (128). Kashiwagi et al. (129) found that in the 123 TNBC cases, the prognosis of patients with E-cadherin-negative expression was markedly worse than that of E-cadherin-positive patients. The Myc oncoproteins (c-Myc, N-Myc, and L-Myc) belong to a family of commonly named “super-transcription factors” that could control the transcription of at least 15% of the entire genome (130). c-Myc overexpression and Myc dependent gene signatures are features of TNBC (131). In breast cancer, carcinoembryonic antigen (CEA) and cancer antigen 15–3 (CA15-3) have been the two most widely used serum tumor markers in the clinical fields for more than 30 years (132). The MUC1 gene is overexpressed in human malignant breast tumors, allowing the use of gene product CA 15-3 as a tumor marker for breast cancer (26). CA15-3 is a monoclonal antibody-defined tumor marker (133) and carcinoembryonic antigen (CEA) is an oncofetal glycoprotein, a widely used tumor marker due to its high expression in adenocarcinoma (134). CEA levels are up-regulated in TNBC patients and the post- Neoadjuvant Chemoradiotherapy (NCRT) CEA plasma levels may be a potential prognostic factor for Disease-Free Survival (DFS), locoregional recurrence-free survival (LRFS) and distant metastasis-free survival (DMFS) in TNBC patients after received NCRT (135). The most studied and reliable biomarkers of CMT are Ki-67, EGFR, HER-2, ER, PR, and COX-2, detected in both serum and tissue samples using different molecular methods. Ki-67 expression is the strongest in CMTs with poor clinical and histopathological characteristics (26). Manuali et al. (136) studied the immunohistochemical expression of CA 15–3 in 7 canine mammary cancer cell lines and 50 malignant mammary tumors and found that CA 15–3 is expressed in both canine mammary tumor cell lines and tissues and that serum levels are significantly correlated with the histological grade. In canine mammary carcinomas, loss of HER2 expression has been associated with a poor prognosis in combination with ER-negative status and positivity of basal cell markers (P-cadherin, p63, cytokeratin 5) (137).

-**Cancer-Associated Stroma (CAS)** α-smooth muscle actin (α-SMA) is the most common marker for detecting cancer-associated fibroblasts (CAFs). CAFs may enhance TNBC progression by activating TGF-beta (138). Until now, understanding of fibroblast activation and ECM remodeling in CMTs have focused primarily on aSMA-positive myofibroblasts, expression of Tenascin-C (Tn-C), MMPs, and their inhibitors (94). Breast cancer cells secrete factors that propel macrophages toward M2 differentiation. The stromal cells also involve resident adipocytes. Leptin, an adipokine, preserved cancer stem cell-like properties in TNBC cells and facilitated tumor recurrence and metastasis. Endothelial cells are well studied in breast cancer and vascular endothelial growth factor (VEGF) significantly dysregulates TNBC (138). Ettlin et al. (139) showed that the underlying biology of CAS is highly comparable between dogs and humans, at least in some aspects, and that COL1A1, ACTA2, and FAP can be used as markers for CAS in canine mammary carcinomas. They also studied the increased expression of Caveolin-1 (Cav1) and FGF2 in CAS. Potential biomarkers for canine and human mammary carcinoma may be EMT-related genes such as COL11A1, COL8A2, and ADAM12 which are overexpressed in mammary carcinoma (95). Matsumoto et al. (140) performed immunohistochemical staining of CD4 and CD8 on tissue microarrays of 164 TNBC cases. Tumor-infiltrating lymphocytes TILs were counted separately as intratumoral (iTILs) and as stromal (sTILs). On Kaplan-Meier analysis, a significantly better survival rate was observed in high CD8 + iTIL and both high CD4 + iTILs and sTILs. Carvalho et al. (141) showed that similar to human breast cancer, macrophages also polarize toward the M2 phenotype in canine mammary cancer and T-lymphocytes, macrophages, and COX-2 share roles in canine mammary carcinogenesis. The severity of lymphocytic infiltrate and the CD4+/CD8+ ratio may represent significant survival prognostic biomarkers for canine mammary carcinomas.

### Circulating Tumor Cells and Tumor DNA (ctDNA)

Tumors release parts of themselves into the circulation, and liquid biopsy is used to analyze circulating tumor cells, circulating tumor DNA (ctDNA), and tumor-derived exosomes (142). Liquid biopsies are commonly blood and urine samples (143). In solid tumors, ctDNA can be shed by necrosis, autophagy, active shedding, and other physiologic events induced by microenvironmental stress and pressure from cancer treatment, as well as normal cell turnover (144). Circulating DNA bears genomic and epigenomic tumor mutational changes, like point mutations, degree of genomic integrity, genomic sequence rearrangements, copy number variation (CNV), microsatellite instability (MSI), loss of heterozygosity (LOH), and DNA methylation (145). Shang et al. (146) who used droplet digital (ddPCR) of circulating free DNA (cfDNA) to determine the PIK3CA mutation status of 49 patients with early-stage TNBC revealed that PIK3CA mutations are associated with relapse-free survival and breast cancer-specific survival and observed that PIK3CA mutations in TNBC are related to androgen receptor phosphorylation, which is known to be an independent prognostic factor for TNBC. Beffagna et al. (147) identified Bcl-2, Bax, and Bad expression in the 78 CMTs using CF41 cells (canine mammary carcinoma cell line, ATCC CRL-6232) by performing protein extraction, western blotting, and immunohistochemistry (IHC). Bcl-2 was expressed more in malignant tumors than in healthy tissue and in benign tumors, as already recorded in CMTs and in several human tumors. Researchers also performed quantitative PCR of plasma cfDNA fragments and found that the neoplastic subjects contained a greater amount of both short and long cfDNA fragments than the non-neoplastic diseased and healthy controls that were again in agreement with previously published data mainly in humans.

### Exosomes and miRNAs

Exosomes are vesicles with a diameter of approximately 100 nm consisting of a lipid bilayer and can be largely classified into membrane components and encapsulated molecules (148). Exosomes can be isolated from plasma, saliva, urine, and cerebrospinal fluid as well as from serum (142). Exosomal proteins including fibronectin, surviving, HER2, periostin, and CD47 have been used as markers for the diagnosis of breast cancer (149). Noncoding RNAs (ncRNAs) do not encode a protein, but rather modulate chromatin regulation and gene expression. They include ribosomal RNAs (rRNAs), transfer RNAs (tRNAs), small nuclear RNAs (snRNAs), small nucleolar RNAs (snoRNAs), microRNAs (miRNAs), small interfering RNAs (siRNAs), piwi-interacting RNAs (piRNAs), and long non-coding RNAs (lncRNAs) (150). Circular RNAs (circRNAs) are a type of non-coding RNA with a closed-loop structure and, are primarily classified into three groups, exonic circRNAs, intronic circRNAs, and exon-intron circRNAs (151). Expression levels of a markedly upregulated circRNA, circGFRA1, were detected in TNBC cell lines and tissues by quantitative real-time PCR (qRT-PCR) (152). Serum exosomal miRNAs, miR-101, and miR-372 can be used as breast cancer diagnostic biomarkers, and serum exosomal miR-373 is indicative for the diagnosis of Triple-negative breast cancer (153). Paszek et al. (154) found that expression of miR-182-5p, and miR-135b-5p was significantly increased while that of miR-190a, miR-136-5p, and miR-126-5p was significantly reduced in TNBC tissues in comparison with normal breast tissues. The miR-199a-5p and miR-342 may be diagnostic markers for TNBC. The miR-93 may be a biomarker linked to TNBC’s biological and clinical characteristics (155). Micro-RNAs can help in early diagnosis, prognosis, and effective treatment for human breast cancer and canine mammary tumor (156). MicroRNA-10b, miR-15a, miR-19a, miR-26b, miR-30a, miR-30c, miR-125a, miR-125b, miR-148a, miR148b, miR-195 and miR-320 are down-regulated both in dogs and in humans while miR-494 is upregulated in both species (157).

### Proteomics and Metabolomics

Klopfleisch et al. (158) used quantitative RT-PCR to identify transcriptional or post-transcriptional regulation of protein expression in metastasizing CMTs and, included 21 proteins with significant changes. 19 of those 21 proteins were previously identified in human breast cancer. Li et al. (159) obtained serum samples from 31 TNBC patients and 31 healthy women in southwestern China and implemented an ultra-high performance liquid chromatography-high resolution mass spectrometry (UHPLC-HRMS) platform for global metabolomic profiling. A substantial percentage of the dysregulated metabolites (45 out of the total 77) were in the class of glycerophospholipids. Using this prognostic information, six metabolites were found to be highly correlated with a 5-year survival rate including dUMP, L-octanoyl carnitine, L-proline, lysophosphatidylcholine (lysoPC), lysophosphatidylcholine (PS), and uric acid. Michishita et al. (160) established metabolite profiles of three canine mammary adenocarcinoma cell lines (CHMp, CNMp, and CTBp) using gas chromatography-mass spectrometry and demonstrated that sphere-forming cells contained increased levels of alanine, glycine, proline, valine, leucine, allo-isoleucine, and isoleucine compared to adherent cells. Valko-Rokytovská et al. (161) found that urinary concentration of tryptophan (TRP), vanillylmandelic acid (VMA), and 3,4-dihydroxyphenylacetic acid (DOPAC) were lower in CMTs and that serotonin (5-HT) and 5-hydroxyindolacetic acid (5-HIAA) concentrations were significantly increased compared with those of healthy control dogs.

**Figure 2:**
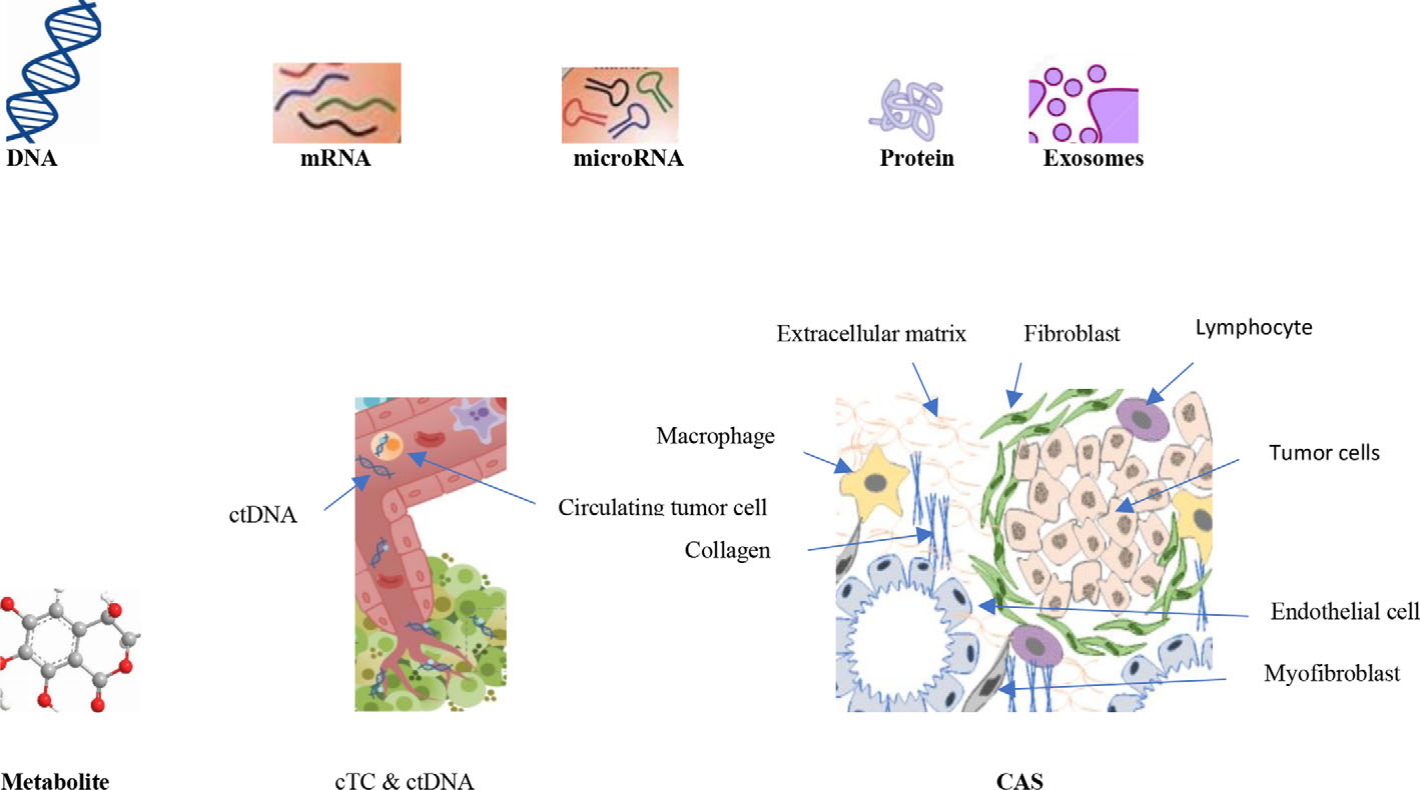
Tumor biomarkers can be DNA, mRNA, microRNA, Protein, Exosome, Metabolites, Circulating tumor cells (cTC), circulating DNA (ctDNA) and Cancer associated stroma (CAS).

TNBC cells exhibit metabolic characteristics expressed by high glycolytic activity and low mitochondrial oxidative phosphorylation (OXPHOS) when compared with hormone-responsive cells. Such metabolic phenotype in TNBC cells may make them highly susceptible to glycolytic inhibition and thus open a window for metabolic interventions specifically targeting TNBCs (162). However both increased and decreased OXPHOS activity is detected in TNBC cells, for example it is remarkably elevated in TNBC with RB1 deficiency (163). A fine balance of the development of reactive oxygen species (ROS) and the ability of production and of an antioxidant system to scavenge ROS are essential for normal cellular functions (164). Moderate levels of ROS and reactive nitrogen species (RNS) can serve as signals for promoting cell proliferation and survival, while severe increases of ROS/RNS can cause cell death (165). Ciani et al. (166) demonstrated that an aqueous extract from Uncaria tomentosa (UT-ex) decreased the dose and time-dependent viability of epidermal squamous cell carcinoma cells, and this delay in cell growth was associated with the increase of reactive oxygen species (ROS). Among the enzymes, superoxide dismutase (SOD), catalase (CAT) and glutathione peroxidase (GPx) is the most important endogenous antioxidants (167). Increased expression of glutathione is observed in canine mammary tumors without ulceration, not metastatic tumors, and low mortality (168). SODs are widespread metalloproteins that act as the most important mechanisms of defense against ROS (169). SOD mimics have a growing therapeutic capability in oncology. These compounds can be used in nontumoral conditions and in conjunction with chemotherapy and radiotherapy to increase the effectiveness of therapy in cancer cells (170). Andreani et al. (171) found that Cu-ZnSOD activity and expression in canine mammary tumors increased substantially compared with healthy control tissues. In a study in domestic cats, the enrichment of the ovary transport medium with SOD decreased cellular apoptosis and improved cumulus-oocyte complexes (COC) survival and *in vitro* embryo production (IVEP) (172). Sarmiento-Salinas et al. (173) observed increased levels of ROS in cell lines of triple-negative breast cancer (TNBC) and dependency on ROS for survival as antioxidant treatment induced cell death in TNBC cells but not in an estrogen receptor-positive (ER+) cell line. The interaction of TNBC cells with CAS leads to the altered metabolic phenotypes in stromal cells and tumor cells such as metabolic interaction with tumor-associated macrophages (TAMs), cancer-associated fibroblasts (CAFs) and cancer-associated adipocytes (CAAs). This metabolic reprogramming of CAS influences metastasis and chemoresistance of TNBC (174) (**Figure 2** and **Table 5**).

**Table 5:** Some major and novel biomarkers in triple-negative breast cancers (TNBC) and canine mammary tumors (CMTs).

Biomarker	TNBC	CMT
ER, PR, and HER2	negative ER, PR, and HER2	The majority are negative for ER, PR and HER2
Ki-67 index	relatively higher in TNBC than in non-TNBC	Ki-67 expression is the strongest in tumors with poor clinical and histopathological characteristics
CK5/6	The most important and relevant marker for defining the basal subgroup of TNBC	Positive in basal-like type of triple negative CMT
VEGF	Intratumor and serum levels remarkably elevated in TNBC compared to non-TNBC	A powerful angiogenic factor in CMT
COL1A1, ACTA2, FAP	Used as markers of CAS in CMTs and human breast carcinoma	
COL11A1, COL8A2, and ADAM12	EMT-related genes overexpressed in HBC and CMT	
α-SMA	may enhance TNBC progression and ECM remodeling in CMT	
CD8+ and CD4+ TILs	good prognostic indicators in TNBCs and CMTs	
Circulating tumor DNA	greater amount of both short and long circulating free DNA fragments in CMTs and HBC	
miRNAs	expression of miR-182-5p, and miR-135b-5p significantly increased and miR-190a, miR-136-5p, and miR-126-5p significantly reduced in TNBC tissues. MicroRNA-10b, miR-15a, miR-19a, miR-26b,miR-30a, miR-30c, miR-125a, miR-125b, miR-148a, miR148b, miR-195 and miR-320 down-regulatedboth in dogs and in humans and miR-494 upregulated in both species	
Exosomes	Exosomal proteins fibronectin, surviving, HER2, periostin, and CD47 used as markers for the diagnosis of breast cancer. Serum exosomal miR-373 indicative for the diagnosis of Triple-negative breast cancer.	
Metabolites	dUMP, L-octanoyl carnitine, L-proline, lysoPC, PS, and uric acid highly associated with 5-year survival rate in TNBC	Decreased urinary concentration of tryptophan (TRP), vanillylmandelic acid (VMA), and 3,4-dihydroxyphenylacetic acid (DOPAC) and remarkably increased urinary concentration of serotonin (5-HT) and 5-hydroxyindolacetic acid (5-HIAA)

## Conclusion

There are many clinical, morphological, and molecular similarities between TNBCs and CMTs. Genomic, proteomic, and metabolomic technologies have enabled the development of exposure, early detection, risk, prognosis, and treatment biomarkers. Multiplex platforms allow many different biomarkers to be analyzed simultaneously and, are therefore attractive screening tools. For instance, LabChip^®^ technology in conjunction with the 2100 Bioanalyzer (Agilent Technologies, Palo Alto, CA, USA) enables the analysis of DNA, RNA, protein, and cellular substances from a single sample (96). The increased number of similarities between human and canine species confirms the hypothesis that the canine mammary cancer cell lines should be regarded as a reliable *in vitro* model for breast cancer research. Establishing an experimental animal model for human breast cancer research would boost the testing of alternative anti-cancer therapies and the development of successful therapies to avoid cancer chemoresistance or multiple drug resistance (68).

## Author Contributions

AN, Dd’A, FC, LA, AG, ML, CI, and FN conceived and designed the review. AN, Dd’A, FC, CI, and FN wrote the review. AG, LA, and ML supervised and guided the entire project. All authors contributed to the article and approved the submitted version.

## Funding

Funds of University of Naples Federico II CdA, n. 52 of 07/29/2019, about the MOU between the College of Science and Technology - Temple University of the Commonwealth System of Higher Education (USA) and the Department of Veterinary Medicine and Animal Production of the University of Naples Federico II (Italy). Funds of University of Naples Federico II, D.R. n. 2564 del 25.06.2019, for International Exchange Program with Foreign Universities for the Short-term Mobility of Teachers and Scholars, between the Temple University of the Commonwealth System of Higher Education (USA) and the University of Naples Federico II (Italy).

## Conflict of Interest

The authors declare that the research was conducted in the absence of any commercial or financial relationships that could be construed as a potential conflict of interest.
